# Disclosure and help seeking behavior of women exposed to physical spousal violence in Dhaka slums

**DOI:** 10.1186/s12889-016-3060-7

**Published:** 2016-05-10

**Authors:** Kausar Parvin, Naznin Sultana, Ruchira Tabassum Naved

**Affiliations:** Universal Health Coverage, Health Systems and Population Studies Division, icddr,b, 68 Shaheed Tajuddin Ahmed Sharani, Mohakhali, Dhaka, Bangladesh; Statistics Department, Head office, Bangladesh Bank, Dhaka, Bangladesh

**Keywords:** Physical spousal violence, Disclosure, Help seeking behavior, Slum

## Abstract

**Background:**

Despite high prevalence of intimate partner violence (IPV) and its adverse social and health consequences, the rate of help seeking for IPV is generally low. Although the level of IPV is much higher in urban slums of Bangladesh, the level and nature of help seeking of the victims are unknown. This paper aims to address this gap in the literature.

**Methods:**

Using a cross-sectional survey conducted between August 2011-February 2012, we explored disclosure of violence, help seeking behavior, and their correlates among randomly selected currently married women aged 15–29 in Dhaka slums (*n* = 2604).

**Results:**

About 60 % of the currently married women reported past year spousal physical violence, but only 21 % disclosed and 19 % sought any help. High acceptance of violence was the main reason for not seeking help. Help was most commonly sought from informal sources (89 %). Any education, frequent and severe physical abuse, and presence of children increased the likelihood of disclosure and help seeking. Most survivors from slum who disclosed also sought help.

**Conclusions:**

Despite widespread physical abuse, many survivors never sought help. Wide acceptance of violence hampering help seeking needs to be challenged. Increasing disclosure would also enhance help seeking. Awareness rising regarding rights of women to live a violence free life is essential. Although many services are available in the urban area, information about these services needs to be available to women. Promoting education is important in increasing both disclosure and service uptake.

## Background

Intimate partner violence (IPV) is a serious public health issue [1–3]. IPV was ranked 23^rd^ for leading attributable risk factors to the global disability-adjusted life years in 2010 [4, 5]. The global lifetime prevalence of IPV is 30 %, with a rate of 42 % in South Asia [6]. Between 13 and 61 % ever-partnered women experience physical IPV across the world [1]. IPV has a wide range of physical and mental health consequences for abused women [7–10] and adverse implications for their children [11–13]. It accrues substantial cost to the nation [14]. Despite high prevalence of IPV and its adverse health outcomes, the rates of disclosure of violence and help seeking are generally low [15–17]. There is a huge variation in the rate of disclosure from country to country (between 21 and 66 %) [18]. Abused women in developing countries mostly seek help from informal sources [15, 16, 19, 20]. In different developing countries, between 55 and 95 % abused women never sought help from any formal institutions [18]. Common reasons for low help seeking are: considering violence as normal or not serious enough for reporting; shame and embarrassment in disclosure, fear that reports would not be believed, and fear of adverse consequences [15, 20, 21].

### Violence against women in Bangladesh

Violence against women (VAW) is ubiquitous in Bangladesh and women report high levels of physical abuse [22, 23]. The national rate of lifetime spousal physical violence was 49 % in 2007 as reported by ever-married women [23]. In urban non-slum areas of Bangladesh, the rate of lifetime physical violence was 46 % in 2006 with an even higher rate in the slums (62 %) [24]. Despite high prevalence rates of IPV in the country, the rate of disclosure and help seeking is quite low. An earlier study by Naved et. al. [15] reported that 66 % of the physically abused rural and urban women never shared their experience with others. Approximately 60 % of the urban and 51 % of the rural abused women seeking help never received a positive response.

### Correlates of disclosure and help seeking

The factors associated with disclosure and help seeking behavior of physically abused women are important for policy and programmatic purposes. Disclosure and help seeking behavior of abused women are shaped by multiple factors, such as severity of violence [10, 15, 17, 19, 25], age of the abused women [17, 26], woman’s income earning status [26], and education [27]. Presence of children in a violent relationship was also associated with help seeking [28]. Women’s perception of violence as normal act as an important barrier in help seeking [29].

Most of the literature on disclosure and help seeking comes from developed countries. The few studies from developing countries on this topic are confined to the general population in urban and rural areas. Thus, despite the fact that the rate of violence is higher in urban slums, little is known about disclosure and help seeking behavior of abused slum women. This impedes formulation of appropriate policies and interventions for supporting these women, who are more vulnerable to physical violence compared to their urban and rural peers. This paper addresses this gap in the literature by looking at the magnitude of physical spousal violence against young women in Dhaka slums, their help seeking patterns, and correlates of disclosure and help seeking.

## Methods

### Design and procedure

The data for this analysis come from a cross-sectional baseline survey of women aged 15–29 carried out in nineteen slums in three different areas of urban Dhaka between August 2011 and February 2012. The survey was conducted as part of a baseline for an action research project, “Growing up Safe and Healthy (SAFE): Addressing Sexual and Reproductive Health and Rights and Violence against Women and Girls in urban Bangladesh.” The details of the study methodology were described elsewhere [30]. Briefly, the study was a three-level multi-site cluster randomized trial. In each site, the clusters were randomly assigned to the three intervention arms. In this design, individuals were nested within clusters and the clusters within each site. A total of 234 clusters (78 clusters per site) were formed, comprising of 186 households on average. Clusters were randomly designated for drawing female and male samples. From each female cluster, 27 individuals were randomly selected for interview. The total number of successfully interviewed women was 4458. A subsample consisting of 2604 women currently married and living with husbands with complete data on physical violence was included in this analysis.

The data collection team comprised of 25 data collectors, with five supervisors and one coordinator. The team received a 13-day training on Gender, Sexual and Reproductive Health and Rights, Violence against Women and Girls (VAWG), research ethics, the survey questionnaire, and survey software. The data were collected through face-to-face household interviews using netbook computers in the offline mode and uploaded to a designated server at the end of each business day.

### Ethics

The WHO ethical guidelines for conducting research on violence against women were followed [31]. Data were collected only after receiving informed consent from each participant. Interviews were conducted in private and in a non-judgmental manner. Participants were referred to appropriate NGOs or government facilities, when needed. The study received ethical approval from icddr,b and Population Council’s Institutional Review Boards.

### Measurement of study variables

#### Dependent variable

Disclosure and help seeking behavior of the physically abused women were treated as dependent variables in the analyses. The physically abused women were asked whether they disclosed their experience of physical abuse to anyone during the last 12 months and responses were coded as ‘yes’ or ‘no.’ They were also asked whether they sought any help from relatives, neighbors, local leaders/leader’s wives club, NGO, legal service provider, police, doctor, pharmacist/compounder, religious healers (*pir/fakir/kabiraj)* or from others and responses were coded as ‘yes’ or ‘no.’

#### Independent variable

A woman’s age, level of education, employment status, household socioeconomic status and presence of children were included in the models as independent variables. Socioeconomic status was measured using information on household assets (e.g., television, radio, fan), land ownership, and dwelling characteristics (source of drinking water and sanitation). Each asset was assigned a weight generated through principal component analysis and scores were standardized in relation to a normal distribution. Each asset was assigned a score and the scores were added for deriving a total score for each household. All individuals were ranked according to the household socioeconomic score. The total sample was divided into quintiles from one (lowest) to five (highest).

A modified version of the Conflict Tactics Scales (CTS) [32] was used to measure physical violence in the past 12 months. In the analysis, frequency and severity of physical violence were also included as independent variables. Frequency of physical violence was categorized as ‘once or few times’ and ‘many times’ in the past 12 months. Physical violence was considered as severe if the women reported being hit with a fist/punched, or kicked/dragged/beaten up, or threatened, or used weapon in the past 12 months. While non severe physical violence included slapping and pushing/shoving in the past 12 months.

Factor analysis was performed to obtain a score for controlling behavior imposed by spouse using seven statements about this behavior. The statements were: (1) Tries to keep you from seeing your friends, (2) Tries to restrict contact with your family of birth, (3) Insists on knowing where you are at all times, (4) Ignores you and treats you indifferently, (5) Gets angry if you speak with another man, (6) Is suspicious that you are unfaithful, and (7) Expects you to ask his permission before seeking health care for yourself. Responses were coded as ‘yes’ or ‘no’. The measure was internally consistent (Cronbach’s alpha = 0.71 and KMO measure = 0.80). The factor score ranging between 0 and 3 was ‘exposed to high level of control’, while a score between −3 and 0 was coded as ‘exposed to low level of control’.

Women’s violence condoning attitudes were also measured using the questionnaires from the WHO multi-country study on Women’s Health and Domestic Violence against Women [18] and International Men and Gender Equality Survey (IMAGES) [33]. The items for measuring attitudes were: (1) You think that a woman cannot refuse to have sex with her husband, (2) You think that when a woman is raped, she is usually to blame for putting herself in that situation, (3) You think that there are times when a woman deserves to be beaten, and (4) You think that a woman should tolerate violence in order to keep her family together. Responses were coded as ‘strongly agree’, ‘agree’, ‘disagree’, and ‘strongly disagree'. Factor analysis was performed to generate attitudinal score (Cronbach’s alpha = 0.54 and KMO measure = 0.67). A binary variable was derived regarding violence against women, where women with scores 0 and below were categorized as holding highly condoning attitudes and women with scores above 0 as holding the least condoning attitudes.

### Statistical analysis

The survey data were weighted to address oversampling of the women aged 15–19 years taking into account distribution of the sample by marital status in the general population. Descriptive analysis was performed to present background characteristics of the participants, current prevalence of physical violence, disclosure, and help seeking pattern of the physically abused women.

Two separate logistic regression models were run to identify the correlates of disclosure and help seeking. For all statistical analyses, the significance level was set at 0.05. The data were analyzed using STATA.

## Results

### Sample characteristics

A majority of the currently married women were more than 19 years old. About 28 % of the women had no education and more than half of them (57 %) were employed at some point in their life. About 35 % held highly condoning attitudes toward violence (Table 1).

**Table 1:** Background characteristics of currently married women (*n* = 2604), %

Characteristics	*N* (%)^a^
Age, in years (*n* = 2604)	
15–19	438 (16.8)
20–24	1077 (41.4)
25–29	1088 (41.8)
Years of education(*n* = 2604)	
0	726 (27.8)
1–5	1105 (42.5)
>5	1773 (29.6)
Employed (Ever) (*n* = 2604)	1474 (56.6)
Presence of children (*n* = 2604)	2135 (81.9)
Socio-economic status(*n* = 2604)	
Quintile 1 (Lowest)	764 (29.3)
Quintile 2 (Second)	489 (18.7)
Quintile 3 (Middle)	453 (17.3)
Quintile 4 (Fourth)	441 (16.9)
Quintile 5 (Highest)	457 (17.5)
Attitude regarding VAW (*n* = 2506)	
Highly condoning	882 (35.1)
Least condoning	1624 (64.8)

^a^all numbers and percentages are weighted

### Prevalence of physical violence

**Fig. 1:**
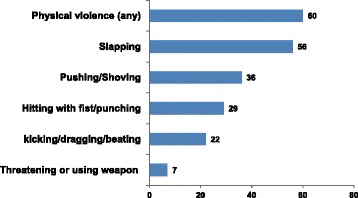
Prevalence of spousal physical violence against women in the past 12 months as reported by women (*n* = 2604), %

The prevalence of current physical violence was 60 % (Fig. 1). The most commonly reported act of physical violence was slapping (56 %). Around 29 % of the abused women were abused frequently (many times in the last 12 months). About 46 % of the abused women reported severe physical violence (Table 2). As a result of physical violence, 26 % women had sustained injuries in the last 12 months and 73 % of the injured women needed health care. The main reason for being physically abused by husband was verbal dispute (53 %) and perceived disobedience of the woman (29 %). Roughly 12 % of the women were abused without any particular reason (Data not shown).

**Table 2:** Severity and frequency of physical violence (*n* = 1566)

Variable	*N* (%)*
Frequency of physical violence	
Once or few times	1119 (71.5)
Many times (frequently)	447 (28.5)
Severity of physical violence	
Non severe	845 (53.9)
Severe	721 (46.0)

*all numbers and percentages are weighted

### Help seeking pattern of the physically abused women

Only 21 % of the physically abused women disclosed their experience and 19 % sought help that depicts almost all of the women who disclosed also sought help. The main reason for not seeking help was high level of violence acceptance (53 %). The other reasons included: concerns about bringing bad name to family (18 %), lack of confidence that this would be useful (16 %), and shame/embarrassment/fear of getting blamed (12 %). Lack of information regarding formal sources of help hindered approximately 14 % of the abused women from seeking help (Data not shown). Those who sought help did so mainly when they were unable to endure violence anymore (80 %).

### Sources of help

Physically abused women most commonly sought help from informal sources, such as relatives from own side (57 %) and neighbors (26 %) (Table 3). A very few women sought help from relatively formal sources, such as local leaders/clubs (5 %) and legal service providers (4 %).

**Table 3:** Sources of help for physically abused women in the past 12 months (*n* = 337), %

*Sources of help*	*N* (%)^a^
*Informal source*	
Relatives from own side	193 (57.3)
Neighbors	86 (25.5)
Relatives from partner’s side	35 (10.3)
Other	6 (1.7)
*Formal source*	
Local Leaders/clubs	17 (5.1)
Legal aid agency/lawyer	13 (3.8)
Police	6 (1.6)
NGO	2 (0.5)
Doctor	0 (0.0)
Compounder/pharmacists	0 (0.0)
Religious healers (*Pir/Fakir/Kobiraj*)	0 (0.0)

^a^all numbers and percentages are weighted

### Correlates of disclosure and help seeking

Two separate logistic regression analyses were performed to identify the correlates of disclosure and help seeking. The same covariates, such as education, presence of children, exposure to high controlling behavior, low VAW condoning attitudes, and exposure to frequent and severe physical violence, were found to be positively associated with disclosure and help seeking. Severe and frequent physical abuse increased the likelihood of disclosure and help seeking. Women who were severely abused were 4.58 times (CI = 3.16–6.66) more likely to disclose and 5.30 times (CI = 3.49–8.05) more likely to seek help compared to the non-severely abused women. The odds of disclosure and help seeking for the women who reported frequent physical abuse in the past 12 months was 1.53 (CI = 1.11–2.19) and 1.83 (CI = 1.28–2.65), respectively. Women with 1–5 years of education were 1.66 times (CI = 1.16–2.46) more likely to disclose and 1.57 times (CI = 1.03–2.39) more likely to seek help compared to women with no education. Women with more than 5 years education were 2.14 times (CI = 1.36–3.36) more likely to disclose and 2.24 times (CI = 1.37–3.63) more likely to seek help compared to women with no education.

Presence of children increased the likelihood of disclosure (OR = 1.78, CI = 1.18–2.69) and help seeking (OR = 1.83, CI = 1.17–2.86). Women who reported experiencing high controlling behavior were more likely to disclose (OR = 1.57, CI = 1.12–2.19) and to seek help (OR = 1.68, CI = 1.17–2.41). Women with low scores in VAW condoning attitudes were more likely to disclose (OR = 1.44, CI = 1.04–1.98) and seek help (OR = 1.42, CI = 1.01–1.99) (Table 4).

**Table 4:** Odds ratio (95 % confidence interval) from logistic regression analyses indentifying correlates of disclosure and help seeking by women physically abused in the past 12 months, *n* = 1522

	Disclosure	Help seeking from any source
Independent variables	Odds ratio(CI^a^)	Odds ratio(CI^a^)
*Age in years*		
15–19 (ref)	1.00	1.00
20–24	0.88 (0.63–1.22)	1.06 (0.75–1.50)
25–29	0.79 (0.54–1.16)	1.80 (0.64–4.99)
*Years of schooling*		
0 years (ref)	1.00	1.00
1 -5 years	1.66 (1.16–2.46)*	1.57 (1.03–2.39)*
More than 5 years	2.14 (1.36–3.36)**	2.24 (1.37–3.63)**
*Ever employed*		
No (ref)	1.00	1.00
Yes	1.08 (0.76–1.53)	1.17 (0.81–1.70)
*Socio-economic status*		
Quintile 1 (ref)	1.00	1.00
Quintile 2	1.39 (0.88–2.17)	1.35 (0.84–2.02)
Quintile 3	1.10 (0.70–1.74)	1.06 (0.66–1.74)
Quintile 4	0.99 (0.65–1.62)	1.07 (0.64–1.78)
Quintile 5	1.04 (0.62–1.74)	0.99 (0.57–1.73)
*Presence of children*		
No (ref)	1	1
Yes	1.78 (1.18–2.69)**	1.83 (1.17–2.86)**
*Controlling behavior*		
Less controlled(ref)	1.00	1.00
Highly controlled	1.57 (1.12–2.19)*	1.68 (1.17–2.41)*
*Violence condoning attitude*		
High VAW condoning attitudes (ref)	1.00	1.00
Low VAW condoning attitudes	1.44 (1.04–1.98)*	1.42 (1.01–1.99)**
*Frequencies of physical violence*		
Once or few times (ref)	1.00	1.00
Many times	1.53 (1.11–2.19)**	1.83 (1.28–2.65)**
*Severity of physical violence*		
Non severe (ref)	1.00	1.00
Severe	4.58 (3.16–6.66)***	5.30 (3.49–8.05)***

^a^Confidence interval

*p = <.05, **p = <.01, ***p = <.001 *Note*, *ref* reference group

## Discussion

Results show that very high proportions (60 %) of the currently married slum women were physically abused during the past 12 months. This rate is much higher than the rate in urban Dhaka (19 % during the last 12 months) reported in Naved et al.[15]. The proportion of women exposed to severe physical violence (46 %) was much higher than reported by ever-married women in urban (19 %) and rural (19 %) Bangladesh [15]. This rate is also much higher than the countries included in the WHO multi-country study on Women’s Health and Domestic Violence against Women except for Peru [18].

Though the rate of current physical violence and its severity in these slums are much higher than in previous studies from Bangladesh and elsewhere not focused on slums, the rates of disclosure and help seeking in the studied slums are much lower [15, 18].

Similar to other studies, we find that the main reason behind low help seeking is high level of acceptance of violence [18]. This is not surprising, given high acceptance of wife beating (36 %) in the society [23]. Women mainly sought help when the violence became unbearable. The findings from the regression model gives an understanding that a combination of severe and frequent physical violence with exposure to controlling behavior makes the women unable to endure the violence anymore and leads to disclose the experience of abuse. This findings are consistent with earlier studies from home [15] and abroad [17, 20, 25], highlighting the importance of frequency and severity of physical violence as correlates of disclosure and help seeking. Additionally, experience of high controlling behavior by husbands positively influence disclosure and help seeking.

Like women from urban and rural Bangladesh [15] slum women also chose to seek help from informal sources rather than from formal sources (e.g. lawyer, police). Though the rate of help seeking from formal source is low (11 %) for slum women, it is still higher than the rate for urban women in general (2 %) [15]. This may suggest a higher agency among slum women.

As identified in many previous studies from different settings, higher education of the abused women was positively correlated with disclosure [34] and help seeking [27]. As pointed out by Cattaneo & DeLoveh [35], this may be due to the fact that higher education leads to better recognition of rights. Presence of children in the violent relationship was also positively associated with disclosure and help seeking, which is similar to findings by Meyer [28]. One possible explanation may be that concerns regarding the well-being of the children make women seek help. This interpretation is supported by the finding from the WHO multi-country study, which shows that in Bangladesh around one-third of the physically abused women sought help because their children were either threatened or hit by the perpetrator [18].

As in other studies, we found that in Dhaka slums, violence condoning attitude precluded help seeking [29]. Despite availability of many more services in Dhaka than in rural areas, abused women preferred to disclose and seek help from family and neighbors, rather than from a formal source. One of the reasons was lack of information about these services (14 % of slum women did not know about such services); however, constraint of resources, such as time and money, may also contribute to this. Valuing the family honor and to avoid embarrassment of disclosure to an outsider, the slum women preferred not to disclose the events of violence to any outsider. This echoes the findings by Montalvo-Liendo [36].

Multiple factors at individual level, household, organizational [34] and community level [37] may contribute toward disclosure and help seeking behavior of the abused women. Community attitudes toward gender-based violence may sometimes discourage the women to seek help [38]. Our findings substantiate this by showing that in the studied slum, high acceptance of violence and high violence condoning attitudes lead to low help seeking. In Bangladesh, the Domestic Violence (Prevention and Protection) Act 2010 created an opportunity to support the abused women, but the extent of use of this law is yet unknown. Evidence is available from different sources that existence of a policy or a law does not necessarily ensure services to a woman. Thus, for instance, in Bangladesh the rate of child marriage was 64 in 2013 [39], despite the provision of The Child Marriage Restraint Act. It is widely recognized that proper implementation of laws is crucial for promoting help seeking of abused women and response to them. Strong informational campaigns for the general population and special training of the legal authority and the law enforcing agencies on available legal provisions for addressing VAW and ways to deal with abused women might improve the services and consequently formal service uptake. Qualitative research will be necessary for gaining deeper insight into the issue enhancing formulation of appropriate interventions and policies.

### Limitation

The cross-sectional design of the study does not allow us to draw any causal inferences. This analysis included only currently married women; thus, women whose relationships ended have not been addressed in this study. By including the currently married women, we covered the majority of ever-married women living in the slum. The analysis was driven only on current prevalence and not those who reported lifetime prevalence. The study findings may not be generalisable. However, study by Naved et al., (15) covering ever-married women and a wider age group (15–49) both in rural and urban areas showed similar correlates of help seeking. Thus, we believe these findings will make valuable contribution to development of policies and programmes.

## Conclusion

This study highlights that a large proportion of women living in urban slums are being physically abused by their husbands and the majority of them have not disclosed or sought any help. A major reason for low help seeking is treating violence as normal, which suggests that attitudes regarding violence need to be changed. Enhancement of disclosure will increase the help seeking rate by abused women. Promoting women’s education is important in increasing disclosure and help seeking by abused women.

### Ethics approval and consent to participate

The study received ethical approval from icddr,b and Population Council’s Institutional Review Boards. Data were collected only after receiving informed consent from each participant.

### Consent for publication

Not applicable.

### Availability of supporting data

The data are available on request to the study Principle Investigator, Dr. Ruchira Tabassum Naved (ruchira@icddrb.org) following the data policy of icddr,b.

## Abbreviations

CTS: conflict tactics scales
IPV: intimate partner violence
NGO: non-governmental organization
VAW: violence against women
VAWG: violence against women and girls
